# Flavonoids compounds from *Tridax procumbens* inhibit osteoclast differentiation by down‐regulating c‐Fos activation

**DOI:** 10.1111/jcmm.14948

**Published:** 2020-01-09

**Authors:** Md. Abdullah Al Mamun, Md. Muzammal Haque Asim, Md. Ali Zaber Sahin, Md. Abdul Alim Al‐Bari

**Affiliations:** ^1^ Department of Genetic Engineering and Biotechnology Shahjalal University of Science and Technology Sylhet Bangladesh; ^2^ Department of Pharmacy University of Rajshahi Rajshahi Bangladesh

**Keywords:** bone resorption, cathepsin K, osteoclast differentiation, *Tridaxprocumbens* flavonoid

## Abstract

The total flavonoids from *Tridax procumbens* (TPFs) have been reported significantly to suppress on RANKL‐induced osteoclast differentiation and bone resorption in mouse primary cultured osteoclasts. However, the effects of ethyl ether fraction of *Tridax procumbens* flavonoids (TPF) on osteoclastogenesis remain unknown. In this study, we investigated the effects of TPF on lipopolysaccharides (LPS)‐induced osteoclast differentiation, actin ring formation, and explored its molecular mechanism in vitro. Matured osteoclast was counted as the number of tartrate‐resistant acid phosphatase (TRAP)‐positive multinucleated cells, and activity of osteoclast was assessed by performing the pit formation assays. Real‐time polymerase chain reaction (RT‐PCR) was performed for evaluation of the expression of osteoclast differentiation‐related genes. TPF reduced the TRAP‐positive multinucleated osteoclasts, inhibited TRAP and acid phosphatase (ACP) activities and decreased the expression of osteoclast differentiating genes, including cathepsin K, metalloproteinase‐2 (MMP‐2), MMP‐9, MMP‐13 and osteoclast‐associated receptor (OSCAR). Furthermore, osteoclast‐dependent actin rings formation and resorption pits were dramatically inhibited by the treatment with TPF. TPF markedly decreased the expression levels of transcription factors such as c‐Fos, nuclear factor of activated T cells cytoplasmic 1 (NFATc1) and activator protein‐1 (AP‐1). Taken together, our findings indicated that TPF suppressed both osteoclast differentiation and activities. Therefore, TPF might be a promising and emerging drug candidate for the treatment of bone diseases such as osteoporosis.

## 1 INTRODUCTION

Osteoclasts are multinucleated bone‐resorbing cells originated from haematopoietic monocyte‐macrophage lineage. Two cytokines, the receptor activator of NF‐κB ligand (RANKL) and the macrophage monocyte colony‐stimulating factor (M‐CSF), are essential for osteoclast formation and activity.^1^, ^2^, ^3^ There are several evidences that low bone mineral density (BMD) is associated with fracture in bone of the older women. Many pathological bone disorders, including postmenopausal osteoporosis, rheumatoid arthritis (RA), osteoarthritis, lytic bone metastasis and Paget's disease are progressed by osteoclast‐induced bone resorption.^4^, ^5^ Thus, identification of small molecules that specifically inhibit osteoclastic activity is a promising strategy of the drug discovery for the treatment of osteoporotic bone diseases.^6^ In addition, bioactive compounds from plant origin have paid emerging attention due to the important sources of potentially useful new therapeutic drugs having less or no side effect.^7^ The *Tridax procumbens* extracts are well‐known phytochemical agents because extracts are used for the treatment of asthma, ulcer, piles and urinary problems.^8^ Previously, we demonstrated that the ethyl ether and ethyl acetate fraction of *Tridax procumbens* flavonoids (TPFs) significantly inhibited osteoclast differentiation and osteoclastic bone resorptive activity. It was reported that the TPFs could inhibit osteoclasts differentiation‐related marker genes expression, including tartrate‐resistant acid phosphatase (TRAP), cathepsin K, matrix metallopeptidase‐9 (MMP‐9) and MMP‐13 in mouse osteoclasts.^9^ Another study investigated that the TPFs induced the differentiation and bone‐forming activity of osteoblasts by enhancing the levels of osteoblast differentiation‐related markers, including alkaline phosphatase (ALP), osteocalcin, type 1 collagen, runt‐related transcription factor (Runx2), osterix, bone morphogenetic protein‐2 (BMP‐2), BMP‐4 and BMP‐7.^10^ Recently, we showed that the only ethyl ether fraction of *Tridax procumbens* flavonoids (TPF) was significantly induced higher bone mass by increasing bone mineral density and bone mineral content in low calcium diet mice model compared with control.^11^ In that report, bone formation parameters, bone volume/tissue volume (BV/TV), number of osteoblast (N.Ob), osteoblast surface/bone surface (Ob.S/BS), mineralizing surface/bone surface (MS/BS), mineral apposition rate (MAR) and bone formation rate (BFR) were enhanced in TPF‐treated mice compared with control.^11^ Moreover, TPF stimulated synergistic effects on BMP‐2–induced bone formation in critical‐sized calvarial defect mouse model.^12^ However, the effect of TPF on differentiation and activity of osteoclast in bone resorption remains uncleared. In the present study, the effects of TPF on lipopolysaccharide (LPS)‐induced osteoclastic differentiation and activities in bone resorption have been investigated. The LPS‐induced osteoclast formation was counted by the number of TRAP‐positive multinucleated cells (two or more nuclei observed under light microscopy). Both TRAP and acid phosphatase (ACP) activities were measured for assessing the role of TPF in osteoclasts differentiation. This is the first evidence in our knowledge that TPF inhibited LPS‐induced osteoclastogenesis by down‐regulating the expression of osteoclasts differentiating markers including TRAP, ACP, cathepsin K, matrix metallopeptidase‐2 (MMP‐2), MMP‐9, MMP‐13, osteoclast‐associated receptor (OSCAR), tumour necrosis factor‐α (TNF‐α, c‐fos, RANKL, nuclear factor of activated T cells cytoplasmic 1 (NFATc1) and activator protein‐1 (AP‐1) as well as destructing the actin ring formation.

## 2 MATERIALS AND METHODS

### 2.1 Chemicals and reagents

α‐Minimal essential medium (α‐MEM), penicillin, streptomycin, foetal bovine serum (FBS), qPCR SuperMix UDG kit were purchased from Invitrogen (Carlsbad, CA). Lipopolysaccharides (LPS), macrophage monocyte colony‐stimulating factor (M‐CSF), methylthiazolyldiphenyl‐tetrazolium bromide (MTT), dimethyl sulfoxide (DMSO), TRAP solution, Acid Phosphatase Liquicolor Assay kit, Hoechst 33342, ELISA kits, alamar blue reagent and phalloidin conjugate solution were purchased from Sigma‐Aldrich. Anti‐NFATc1 (H‐110), anti‐Ap1 (Sc‐57761) and anti–c‐Fos (H‐125) monoclonal antibodies were purchased from Santa Cruz Biotechnology. Anti–TNF‐α, anti‐RANKL, anti‐OPG, anti–IL‐1β, anti–IL‐6 and anti–TGF‐β were purchased from Cell Signaling Technology. NucleoSpin for RNA extraction kit was purchased from Macherey–Nagel. Toluidine blue stain was purchased from BioGenex. FastStart Essential DNA Green Master was purchased from Roche. Goat anti‐rabbit horseradish peroxidase conjugate was purchased from Bio‐Rad.

### 2.2 Selection of the plant material

*Tridax procumbens* plant is widely available in Bangladesh which was identified and authenticated by Professor Dr Anwar Ul Islam, Department pharmacy, University of Rajshahi, Bangladesh. The samples were collected from the Laccatura Tea State, Sylhet, Bangladesh, and sample specimen was submitted to the Department of Genetic Engineering and Biotechnology of Shahjalal University of Science and Technology, Sylhet‐3114, for voucher reference number GEB09032014/3 and processing for sample investigation.

### 2.3 Sample preparation

The different parts of *Tridax procumbens* such as leaf, root and flowers were collected, shade dried and crushed for finely powdered form as detailed explained elsewhere with minor modifications.^8^ In brief, finely powdered samples (200 g in each) were extracted with 80% methanol in Soxhlet distillation and filtrates were repeatedly extracted with petroleum ether, ethyl ether and ethyl acetate by using separating funnel. Petroleum ether fraction was discarded as being rich in fatty substances, whereas the ethyl ether fraction that contains free flavonoids and ethyl acetate fraction that contains flavonoids with bounded sugars. For this present study, we collected the ethyl ether fraction flavonoids from separation funnel, dried and used for osteoclast differentiation.

### 2.4 Total flavonoids determination

The flavonoid content was estimated by aluminium chloride (AlCl_3_) method as described elsewhere with some modifications.^8^ The dried ethyl ether fraction flavonoids were separately mixed with methanol (1.5 mL), aluminium chloride (0.1 mL of 10% AlCl_3_), potassium acetate (0.1 mL of 1 mol/L) and distilled water (2.8 mL) and kept at room temperature for 0.5 hour The absorbances were measured with a spectrophotometer (at 415 nm) and compared with quercetin as a standard. Finally, the flavonoids were dissolved with 75% methanol for further use.

### 2.5 Osteoclast differentiation assay

For isolation of primary bone marrow cells (BMCs), C57BL/6 female mice (average eight‐week‐old and weight 40 g) were killed with the help of CO_2_ and mice femur and tibia were dissected aseptically. The BMCs were flushed out with phosphate‐buffered saline (PBS) and α‐minimal essential medium (α‐MEM) medium (Invitrogen). About 1.5 × 10^4^ cells/well were cultured in α‐MEM medium containing M‐CSF (50 ng/mL), LPS (1 ng/mL), foetal bovine serum (FBS) (10%) and antibiotics (100 U/mL of penicillin G and streptomycin 100 ng/mL) followed by treatment with TPF concentrations of 50 and 100 μg/mL and 75% methanol used as control. Medium was replaced every 3 days and after 6 days, cultures were fixed with 4% paraformaldehyde in phosphate‐buffered saline (PBS) for 20 min was added in cells for fixation and TRAP staining according to the manufacturer's instruction. Finally, cells were washed with cold PBS for three times. All the experimental procedures were reviewed and approved by the Institutional Animal, Medical Ethics, Biosafety and Biosecurity Committee, University of Rajshahi, Bangladesh (Memo No. 62/320 IAMEBBC/IBSc).

### 2.6 TRAP and ACP activity assays

TRAP and ACP assays for osteoclast differentiation were performed as described elsewhere with some modifications.^9^ In brief, the TPF‐treated cells were lysed by Trion‐X‐100 in PBS (0.2%), and the supernatant was measured for TRAP activity using para‐Nitrophenylphosphate (pNPP) with a reaction buffer (0.1 mol/L sodium acetate (pH 5.8), 1 mmol/L ascorbic acid, 0.15 mol/L KCl and 10 mmol/L disodium tartrate) and ACP activity using Acid Phosphatase Liquicolor Assays Kit (Sigma‐Aldrich) according to the instruction of manufacturer. About 0.3N NaOH solution was used for stopping the reaction, and optimum densities were measured at 405 nm by using microplate spectrophotometer (T60 U, PG Instruments Ltd.).

### 2.7 Cell viability assay

For colorimetric method, methylthiazolyldiphenyl‐tetrazolium bromide (MTT) (Sigma‐Aldrich) was used for the viability of cells as described elsewhere with little deviation.^11^ Briefly, about 1.4 × 10^7^ cells/well of BMCs seeded in 6‐well plates. After 4 days, culture cells were treated with TPF at different concentrations (50, 100 µg/mL) for 48 hours. Then, fresh medium containing 0.5 mg/mL MTT was added to the cultured cells for 4 hours. The cells produced blue formazan products were dissolved in dimethyl sulfoxide (DMSO) and measured at 550 nm under spectrophotometrically (T60 U, PG Instruments Ltd.).

### 2.8 Osteoclastic bone resorption assay

For assessment, the effects of TPF on osteoclastic bone resorption, pit formation assay in vitro was performed as described previously.^9^ Briefly, the BMCs were cultured in dentin slices, α‐MEM containing M‐CSF (50 ng/mL), LPS (1 ng/mL) and FBS (10%) for 6 days with different concentrations of TPF. Then, the cells were treated with NH_4_OH (1 N) for 5 min and stained with 0.5% toluidine blue. The resorption pit was visualized under a microscope and analysed by image software system (KS400; Carl Zeiss).

### 2.9 Actin ring formation assay

Osteoclastic actin rings were detected by using phalloidin staining assay. Briefly, the cultured cells were fixed with 3.7% formaldehyde in PBS for 10 min. The fixed cells were permeabilized with 0.1% Triton X‐100 for 10 min and stained with phalloidin conjugate solution (Sigma‐Aldrich) for 40 min. Then, the fixed cells were washed with PBS and the nuclei were stained with Hoechst 33342 (Sigma‐Aldrich). Fluorescence microscope was used for photomicrographs and distributions of actin rings (KS400; Carl Zeiss).

### 2.10 Real‐time Polymerase chain reaction assay

The primary osteoclasts were cultured in 6‐well plate for 6 days after different concentration of TPF treatment (50 and 100 μg/mL). NucleoSpin (Macherey–Nagel) was used for the isolation of the total RNA from each well of cells. RNA aliquots were reversed transcript to complementary DNAs by using reverse transcriptase kit (Fermentas). The cDNA products were subjected to PCR amplification with gene‐specific primers (Table 1) for mouse TRAP, ACP, cathepsin K, MMP‐2, MMP‐9, MMP‐13, TNF‐α, RANKL, Osteoprotegerin (OPG), interleukin‐1β (IL‐1β), IL‐6, transforming growth factor‐β (TGF‐β, c‐Fos, NFATc1 and AP‐1 were synthesized by Integrated DNA technology. Real‐time RT‐PCR amplification was performed with a Light Cycler System (Roche) with a Platinum SYBR Green qPCR SuperMix UDG kit (Invitrogen).^9^

**Table 1:** Primer sequences of real‐time PCR

Gene	Forward	Reverse
TRAP	5′ ‐ACAGCCCCCACTCCCACCCT	5′ ‐TCAGGGTCTGGGTCTCCTTGG‐3′
ACP	5′ ‐CATGCAAGCTATTGAGAAAAGTGGT‐3′	5′ ‐CCTTGATATGTACGTGTTTTCTCG‐3′
Cathepsin K	5′ ‐GGAAGAAGACTCACCAGAA‐3′	5′ ‐GTCATATAGCCGCCTCCA‐3′
MMP‐2	5′ ‐CTCAGATCCGTGGTGAGATCT‐3′	5′ ‐CTTTGGTTCTCCAGCTTCAGG‐3′
MMP‐9	5′ ‐CTGGACAGCCAGACACTAAAG‐3′	5′ ‐CTCGCGGCAAGTCTTCAGAG‐3′
MMP‐13	5′ ‐GTCTGAGATTTGTAGGCCG‐3′	5′ ‐3′ TCATCAAGCTTCTGTCTGTGC‐3′
OSCAR	5′ ‐AATGGACCAATCAGCAGGAC‐3′	5′ ‐GAGAACAAAGCTCCCACAGC‐3′
OPG	5′ ‐GCTGAGTGTTTTGGTGGACAGTT‐3′	5′ ‐GCTGGAAGGTTTGCTCTTGTG‐3′
IL‐1 β	5′ ‐AAACAGATGAAGTGCTCCTTCCAGG‐3′	5′ ‐TGGAGAACACCACTTGTTGCTCCA‐3′
IL‐6	5′ ‐CAAGTCGGAGGCTTAAAC‐3′	5′ ‐AAGTGCATCATCGTTGTTCAT‐3′
TNF‐α	5′ ‐TCTCTACCTTGTTGCCTCCTCTTTT‐3′	5′ ‐GTAGGGCAATTACAGTCACGG‐3′
RANKL	5′ ‐ACATCGGGAAGCGTACCTACA‐3′	5′‐GCTCCCTCCTTTCATCAGGTT‐3′
TGF‐β	5′ ‐CCCAGCATCTGCAAAGCTC‐3′	5′ ‐GTCAATGTACAGCTGCCGCA‐3′
c‐Fos	5′ ‐CGGGTTTCAACGCCGACTA‐3′	5′ ‐TTGGCACTAGAGACGGACAGA‐3′
NFATc1	5′ ‐5′ TGGAGAAGCAGAGCACAGAC‐3′	5′ ‐GCGGAAA‐ GGTGGTATCTCAA‐3′
AP‐1	5′ ‐AAC CCA GAG AGG AAA AGA CT‐3′	5′ ‐TGCAGGAAAGGAGAGAGAG‐3′
b‐actin	5′ ‐GTACGCCAACACAGTGCTG‐3′	5′ ‐CGTCATACTCCTGCTTGCTG‐3′

### 2.11 Enzyme‐linked immunosorbent assay (ELISA)

The concentrations of TNF‐α, RANKL, OPG, IL‐1β, IL‐6, TGF‐β, c‐Fos, NFATc1 and AP‐1 were determined by using respective ELISA kits (Sigma‐Aldrich). Briefly, TPF‐treated cells culture medium was collected, centrifuged and piled up for the measurement of TNF‐α, RANKL, OPG, IL‐1β, IL‐6, TGF‐β, c‐Fos, NFATc1 and AP‐1. The samples were taken in monoclonal antibody‐coated 96‐well micro‐titre plates and incubated for 2 hours at room temperature. After washing the samples with washing buffer (50 mmol/L Tris, 200 mmol/L NaCl and 0.2% Tween 20), horseradish peroxidase（HRP）I conjugated streptavidin was added. The absorbances of the samples were determined at 450 nm (T60 U, PG Instruments Ltd.). Results were represented as percent change of activity compared with the control sample.

### 2.12 Western blot analysis

The c‐Fos, NFATc1 and AP‐1 were detected by Western blotting as described elsewhere with some modification.^9^ Briefly, 6 days after the TPF‐treated primary osteoclasts were lysed and the protein concentrations of lysed fractions were determined by Bio‐Rad Protein Assay. For Western blotting, cell lysate proteins (50 mg) were fractionated onto sodium dodecyl sulphate‐polyacrylamide gel electrophoresis (SDS). The proteins were transferred to polyvinylidene difluoride (PVDF) membranes using transfer buffer (50 mmol/L Tris, 190 mmol/L glycine, and 10% methanol) at 100 V for 2 hours. Then, the membranes were incubated with blocking buffer (50 mmol/L Tris, 200 mmol/L NaCl, 0.2% Tween 20 and 3% BSA, bovine serum albumin) overnight at 4°C. After washing three times with washing buffer (blocking buffer without 3% BSA) for 10 minutes each, the blot was incubated with respective primary antibody such as of c‐Fos, NFATc1 and AP‐1 (Santa Cruz Biotechnology) for 2 hours, followed by HRP‐labelled secondary antibody for 1 hour. The membranes were washed three times and scanned with enhanced chemiluminescence system (T60 U, PG Instruments Ltd.).

### 2.13 Statistical analyses

We analysed all data Student's *t* test followed by analysis of variance (ANOVA) with *F* test. *P* values <.05, .001 and .0001 were considered significant, very significant and strongly significant, respectively. The data are represented as mean (m) ± standard deviation (SD) values of independent replicates.

## 3 RESULTS

### 3.1 Effect of TPF on LPS‐induced osteoclast differentiation

**Figure 1:**
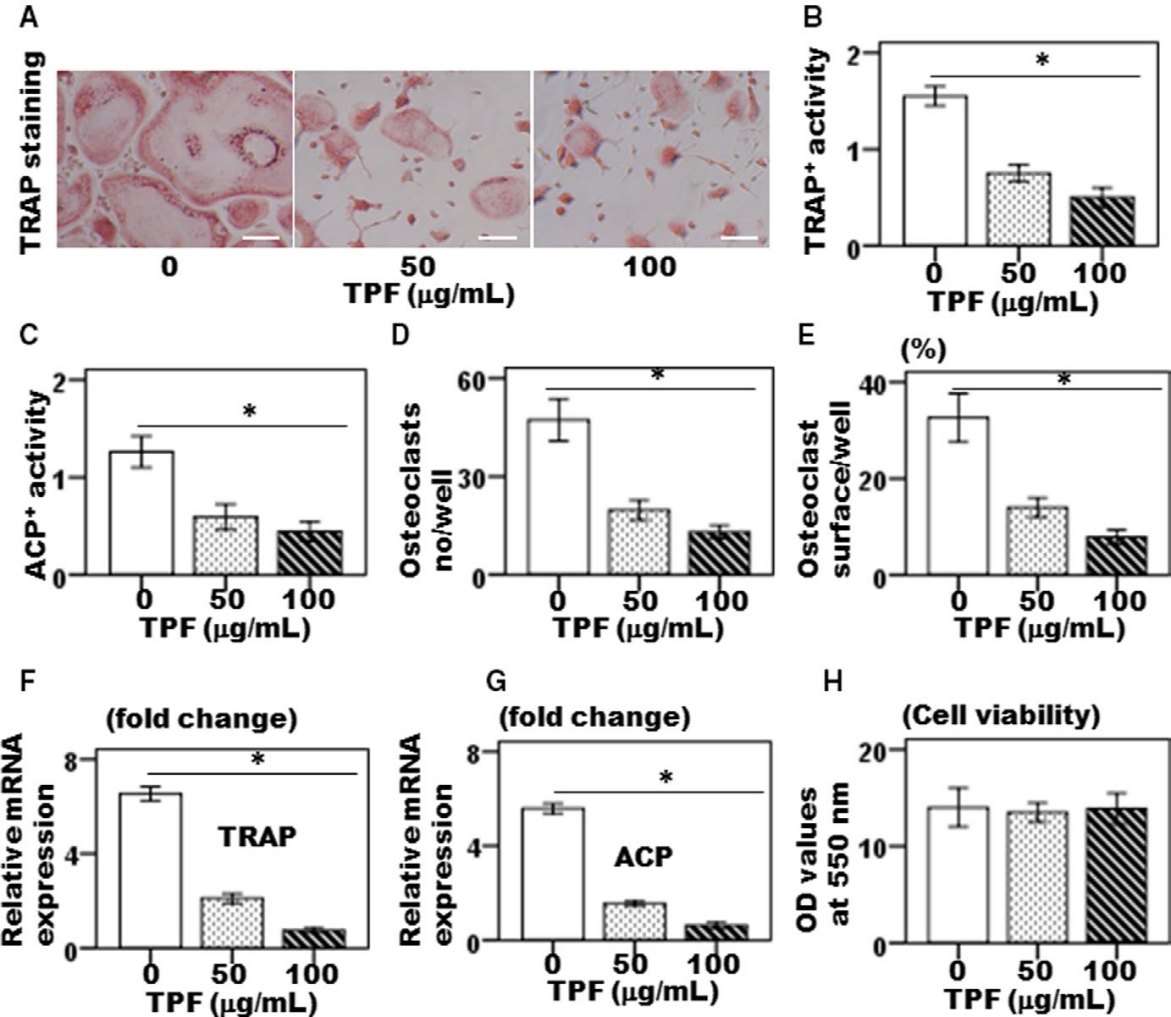
The effects of TPF on LPS‐induced osteoclast differentiation in vitro. The BMCs were cultured with TPF (50 and 100 μg/mL) for 6 d in the presence of M‐CSF (50 μg/mL) and LPS (1 ng/mL) and then evaluated the inhibitory effects of TPF by using TRAP staining activity assay. (A) TRAP^+^ ‐multinucleated osteoclasts are shown in red colour and scale bar represents 50 μm, (B) TRAP activity, (C) ACP activity, (D) osteoclast number/well (N.Oc/well), (E) osteoclast surface/well (Oc.S/well), (F) relative gene expression of TRAP, (G) relative gene expression of ACP and (H) the cell viability counts. The data were represented as mean ± SD (n = 5) of 5 independent experiments. *P < .05 vs TPF of 100 μg/mL

The TRAP‐positive multinucleated osteoclasts (two or more nuclei observed under light microscopy) were significantly decreased dose dependently by TPF treatment (Figure 1A). The TPF‐treated osteoclasts at the concentration of 50 μg/mL were exhibited smaller and fewer nuclei compared with the control group (Figure 1A). Moreover, treatment with TPF at the concentration of 100 μg/mL showed markedly reduced multinucleated osteoclasts in culture compared with the control (Figure 1A). The TRAP and ACP activities were dose dependently decreased in cultured cells that treated with TPF at the concentrations of 50 and 100 μg/mL compared with control group (Figure 1B,C). The osteoclast surface was markedly decreased in TPF‐cultured cells at the concentrations of 50 and 100 μg/mL compared with the control group (Figure 1D,E). RT‐PCR data indicated that TRAP and ACP gene expression levels per well were significantly lower in TPF‐treated cells compared with the control group (Figure 1F,G). Moreover, the TRAP and ACP gene expression levels were substantially lower in cells treated with TPF at higher concentration (100 μg/mL) compared with the cells treated with TPF at 50 μg/mL concentration. So, TPF affected the TRAP and ACP genes expression levels in a dose‐dependent manner. Additionally, cell viability results showed that TPF exposure to higher concentration (100 μg/mL) did not affect the death spots of primary osteoclasts (Figure 1H).

### 3.2 Effect of TPF on LPS‐induced osteoclastic bone resorption

**Figure 2:**
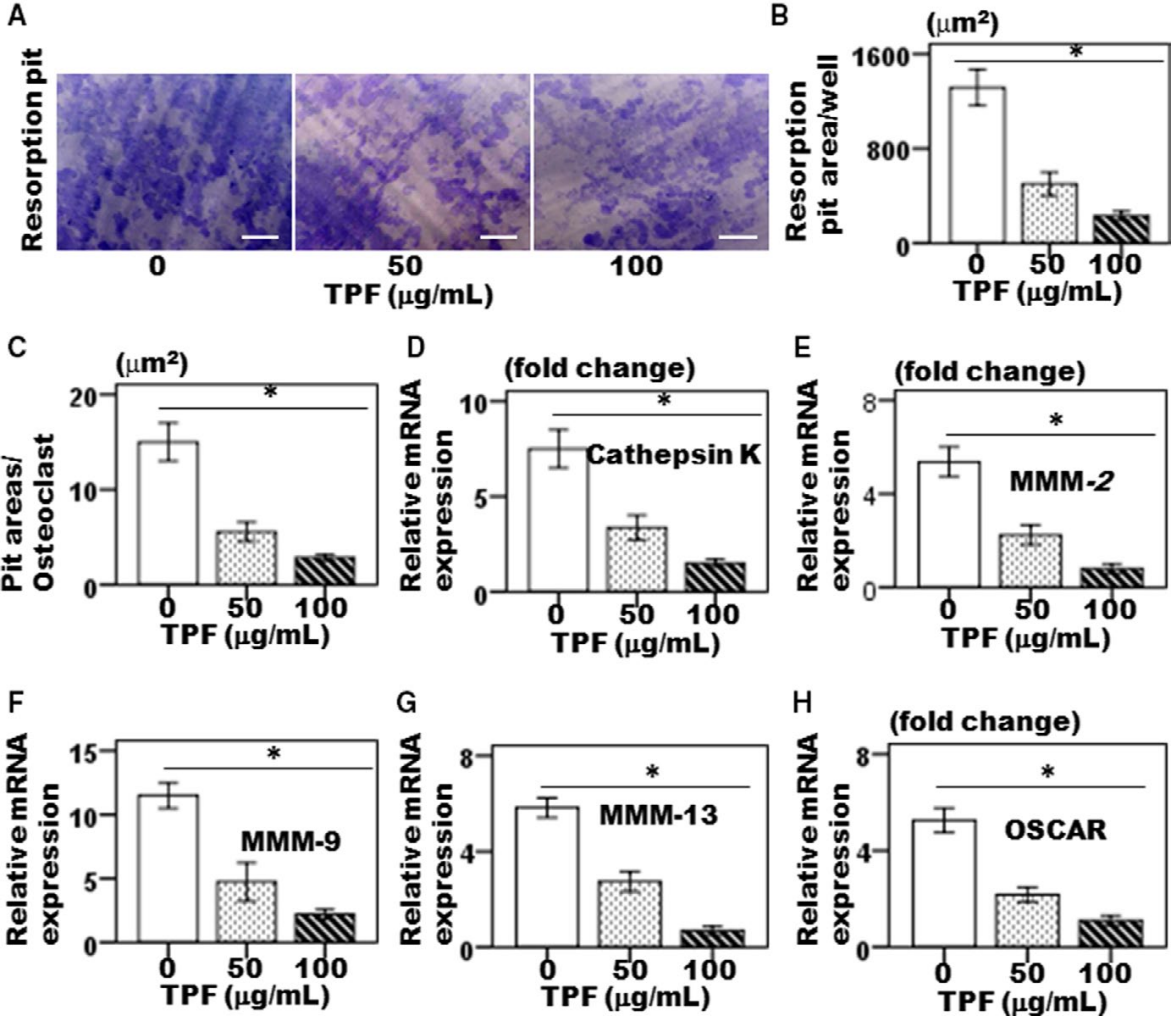
The effects of TPF on LPS‐induced osteoclast activity. The BMCs were cultured with TPF (50 and 100 μg/mL) for 6 days in the presence of M‐CSF (50 ng/mL) and LPS (1 ng/mL), and plates were fixed and stained with toluidine blue. (A) active mature osteoclasts and scale bar represents 50 μm, (B) total resorbed pit area/well, (C) resorbed pit area/osteoclast, (D) Expression of cathepsin K, (E) Expression of MMP‐2, (F) Expression of MMP‐9, (G) Expression of MMP‐13 and (H) Expression of OSCAR. The data were represented as mean ± SD (n = 5) of 5 independent experiments. *P < .05 vs TPF of 100 μg/mL

To examine whether TPF inhibited on LPS‐induced bone resorption activity of osteoclasts, pit formation assay was performed in vitro. LPS‐induced bone resorption was diminished by TPF in a dose‐dependent manner in primary osteoclasts (Figure 2A). TPF dramatically inhibited the density of resorption pits on the osteoclast surfaces (Figure 2B). TPF also diminished the total resorption areas of active osteoclasts (Figure 2B,C). These data suggested that TPF inhibited LPS‐induced bone resorption activity of osteoclasts.

### 3.3 Effect of TPF on osteoclast differentiation‐specific gene expressions

For evaluation, the effect of TPF on expressions of key osteoclast differentiation, specific marker gene levels such as cathepsin K, MMP‐2, MMP‐9, MMP‐13 and OSCAR, RT‐PCR were applied. The BMCs were exposed with TPF at concentrations of 50 and 100 μg/mL. Exposure to TPF suppressed the expression key osteoclast differentiation‐specific marker genes, including cathepsin K, MMP‐2, MMP‐9, MMP‐13 and OSCAR compared with the control group (Figure 2D‐H).

### 3.4 Effects of TPF on the expressions of TNF‐α RANKL and OPG

**Figure 3:**
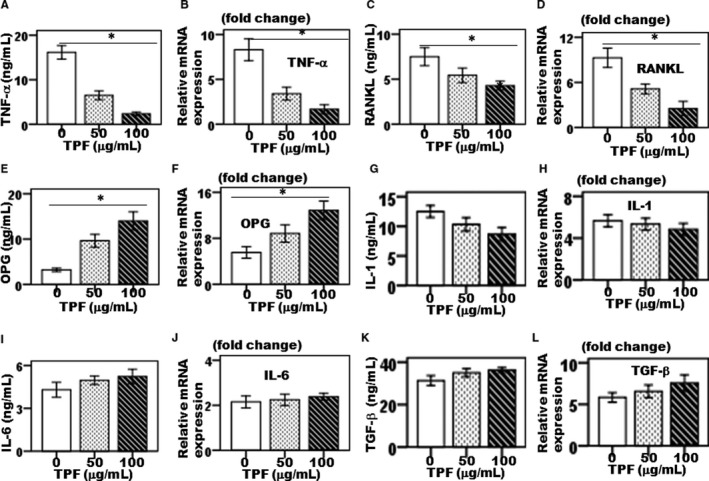
The effects of TPF on bone pro‐inflammatory cytokines synthesis. (A) synthesis of TNF‐α protein, (B) expression of TNF‐α gene, (C) synthesis of RANKL protein, (D) expression of RANKL gene, (E) synthesis of OPG protein, (F) expression of OPG gene, (G) synthesis of IL‐1 protein, (H) expression of IL‐1 gene, (I) synthesis of IL‐6 protein, (J) expression of IL‐6 gene, (K) synthesis of TGF‐β protein and (L) expression of TGF‐β gene. The data were represented as mean ± SD (n = 5) of 5 independent experiments. *P < .05 vs TPF of 100 μg/mL

To address the molecular mechanism of TPF on osteoclastogenesis, we evaluated the TNF‐α, RANKL, OPG, IL‐1β, IL‐6 and TGF‐β expression both in protein and gene levels. Total proteins and mRNA were isolated after the treatment of osteoclasts with TPF at the different concentrations (50 and 100 μg/mL) for 6 days. The results showed that TPF significantly down‐regulated the TNF‐α and RANKL expressions (Figure 3A‐D) and up‐regulated the OPG expression both in protein and gene levels (Figure 3E‐F). This natural compound TPF did not show any notable effects on expressions of IL‐1β, IL‐6 and TGF‐β both in protein and gene levels (Figure 3G‐L).

### 3.5 Effect of TPF on LPS‐induced actin ring formation

**Figure 4:**
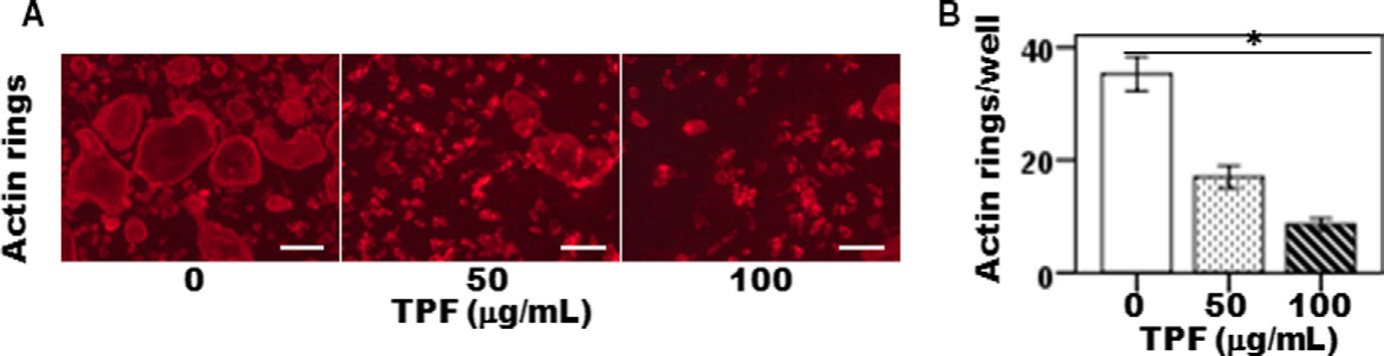
The effects of TPF on actin rings formation. (A) actin rings formation and scale bar represents 50 μm and (B) number of actin rings/well. The data were represented as mean ± SD (n = 5) of 5 independent experiments. *P < .05 vs TPF of 100 μg/mL

We investigated whether TPF could affect on LPS‐induced actin ring formation. In the presence of LPS exposure, primary osteoclasts were differentiated into active osteoclasts and appeared distinct actin ring structures (Figure 4A). However, TPF potentially decreased the number and size of actin rings compared with control group (Figure 4A,B). These results indicated that TPF suppressed on LPS‐induced actin rings and bone resorptive activity.

### 3.6 Effects of TPF on the expressions of c‐Fos, NFATc1 and AP‐1

**Figure 5:**
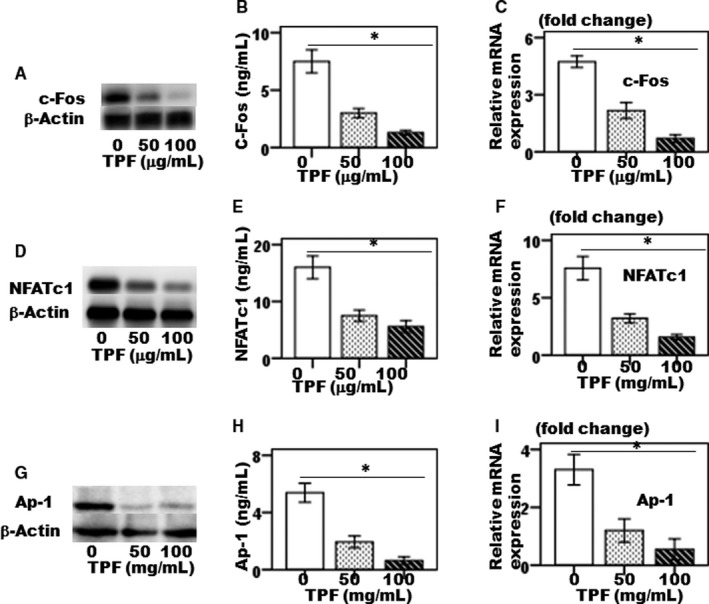
The effect of TPF on LPS‐induced osteoclast‐specific transcription factors. (A, B) c‐Fos protein synthesis, (C) expression of c‐Fos gene, (D, E) NFATc1 protein synthesis, (F) expression of NFATc1 gene, (G, H) Ap‐1 protein synthesis and (I) expression of AP‐1 gene. The data were represented as mean ± SD (n = 5) of 5 independent experiments. *P < .05 vs TPF of 100 μg/mL

It is known that several transcription factors such as c‐Fos, NFATc1 and AP‐1 are essential for osteoclast differentiation. Therefore, cells were treated with TPF at different concentrations (50 and 100 μg/mL) for 6 days in the presence of M‐CSF (50 μg/mL) and LPS (1 ng/mL), and were investigated the TPF effects on LPS‐induced c‐Fos, NFATc1 and AP‐1 expressions in osteoclasts. Treatment of cells with TPF significantly decreased LPS‐induced c‐Fos, NFATc1 and AP‐1 expression both in protein and gene level (Figure 5A‐I), suggesting that TPF inhibit LPS‐induced down‐regulation of the transcription factors c‐Fos, NFATc1 and AP‐1 that required for osteoclast differentiation.

## 4 DISCUSSION

Multinucleated osteoclasts attributed to a bone homeostatic imbalance between bone resorption and bone formation which is common in pathological hallmarks of bone destruction, including osteoporosis, inflammatory joint disorders, rheumatoid arthritis, periodontitis and bone cancer. Therefore, control of osteoclast differentiation is an identified therapeutic strategy to the treatment of osteoclast‐specific diseases.^13^ The drugs are currently used in osteoclast‐specific disease treatment primarily includes steroids, for example oestrogen replacement therapy, selective oestrogen receptor modulators (SERMs) and bisphosphonates, RANKL inhibitors, parathyroid hormone (PTH) peptides, anti‐Dickkopf‐related protein 1 (DKK1), anti‐sclerostin (SOST) antibodies and strontium ranelate (SR) are broadly classified as either bone anabolic or anti‐bone resorptive agents involved bone resorption inhibition, which maintain balance between bone remodelling and bone mass by inhibiting osteoclast differentiation.^13^, ^14^ However, most of these drugs are associated with side effects, including hypertension, thromboembolism, endometriosis and hypercalcemia.^13^ As osteoclasts are responsible for bone destruction‐specific diseases, therefore developing new drugs for therapies that have both bone anabolic and anti‐bone resorption effects would be more effective in treating bone loss in osteoclast‐specific diseases like osteoporosis. Total flavonoids isolated from TPFs are able to inhibit osteoclast differentiation and bone resorption. Previously, we showed that TPFs could suppress RANKL‐induced osteoclast differentiating marker genes expressions such as TRAP, cathepsin K, MMP‐9 and MMP‐13.^9^ We have also reported the anabolic potential of TPFs on osteoblast differentiation and bone formation by enhancing expressions of ALP, osteocalcin, type 1 collagen, Runx2, osterix, BMP‐2 and BMP‐7.^10^, ^11^ In the present study, we examined the effects of TPF on LPS‐induced osteoclast differentiation and bone resorption in primary mouse osteoclasts. We found that TPF decreased LPS‐induced TRAP and ACP activities, and suppressed bone resorption without affecting the osteoclasts viability (Figure 1A‐H), suggesting that the inhibition of osteoclast generation by TPF was not owing to adverse side effect on the cells. Gram‐negative bacterial LPS‐secreted various inflammatory cytokines stimulates fusion stage of pre‐osteoclasts and promotes differentiation of osteoclast.^15^ In this model, we found that TPF diminished on LPS‐induced TRAP, ACP and bone resorptive activities in dose‐dependent manner. In this study, we have reported that TPF is capable to suppress differentiation of osteoclasts and bone resorption, as TPF suppresses the production of TRAP‐positive multinucleated osteoclasts from mouse‐cultured osteoclasts. These data indicate that TPF suppress the production of multinucleated osteoclasts from the fusion of their precursor cells, as a result of reducing the number of nuclei per osteoclasts. Active osteoclasts characterized by the ruffled border membrane and basolateral secretory vesicles. These ruffled borders initiate activation of hydrolase enzymes and provide acidic environment for solubilizing and digesting the bone matrixes.^16^, ^17^ TPF treatment completely disrupted actin ring formation and osteoclastic bone resorption. Moreover, TPF suppressed LPS‐induced osteoclast differentiation‐specific marker genes, including TRAP, ACP, cathepsin K, MMP‐2, MMP‐9, OSCAR and TNF‐α which play an essential role in bone remodelling. TRAP and ACP are enzymes that highly expressed in osteoclasts and contribute pathological bone resorption by prompting the dephosphorylation of bone matrix phosphoproteins such as osteopontin and bone sialoprotein.^18^, ^19^ Both TRAP and ACP are crucially participated in osteoclast formation which directly related to the osteoclastic bone resorption and cartilage destruction. Blockade of TRAP and ACP production during LPS‐induced osteoclast formation is highly potent protector of osteoclastic bone resorption and cartilage destruction.^18^, ^19^ The present data support the concepts that TRAP and ACP are significantly decreased in the TPF‐treated osteoclasts compared with the control group (Figure 1A‐G). It was reported that cathepsin K is essential for making initial structures of actin rings as well as activity of osteoclasts.^17^ Here, our data suggested that TPF efficiently inhibited mRNA expression of cathepsin K and formation of actin rings were completely disrupted at the concentration of 100 μg/mL. Our results demonstrate that TPF plays a pivotal role in controlling osteoclastic bone resorption via diminished actin ring formation and bone resorptive enzymes. It was investigated that OSCAR expressed in bone and cartilage is identified as an important costimulatory receptor for osteoclast differentiation through activation of NFATc1. It also showed that OSCAR participates in the progression of diseases such as osteoporosis and rheumatoid arthritis.^20^ The MMP‐2, MMP‐9 and MMP‐13 are enzymes that play critical role in osteoclastic bone resorption, joint degeneration and inflammatory arthritis progression by possessing the combined ability to degrade the organic components of connective tissue matrices.^21^ In this report, we demonstrated that TPF inhibited LPS‐induced osteoclast formation and significantly decreased expression of MMP‐1, MMP‐3 and MMP‐9 in proteins and genes levels compared with the control group (Figure 2E‐G). Therefore, our results suggest that TPF have potential therapeutic effects associated with the decreased MMP‐1, MMP‐3 and MMP‐9 expressions. Here, we reported for the first time inhibitory effects of TPF on LPS‐induced osteoclast differentiation and bone resorption. Previous evidences showed that pro‐osteoclastogenic cytokines, like TNF‐α and RANKL, are essential and adequate to promote osteoclastogenesis and bone resorption as seen in osteoporosis, postmenopausal and periodontitis, animal model that strongly augment osteoclast lifespan. Therefore, blocking of this pro‐osteoclastogenic cytokines synthesis is proven therapeutic approach to the treatment of bone loss‐related diseases.^22^, ^23^ Our results confirmed that treatment with TPF of LPS‐induced osteoclastogenesis significantly diminished the increase levels of TNF‐α and RANKL (Figure 3A‐D). OPG is a member of the TNF superfamily protein secretes by osteoclasts. OPG negatively regulates RANKL‐induced osteoclastogenesis and bone resorption in vitro and in vivo by binding to RANKL with high affinity that competes with RANK and suppresses by osteoclasts formation.^24^, ^25^ In the present study, we found that TPF inhibited LPS‐induced osteoclastogenesis and subsequently increased the expression of OPG in cultured cells (Figure 3E‐F). The important transcription factors such as c‐Fos, NFATc1 and AP‐1 are essential for osteoclastogenesis. The transcription factor c‐Fos knockout mice show an osteopetrotic phenotype failed to produce osteoclasts, whereas stem cells derived from NFATc1 knockout mice failed to differentiate into osteoclasts.^26^ In this study, TPF essentially inhibited NFATc1 expression levels, as up‐regulation normally saw after LPS treatment. Evidence showed that NFATc1 is regulated by AP‐1, a dimeric transcription factor of members of Jun and Fos protein family.^26^ For NFATc1 stimulation, up‐regulation c‐Fos of is required. If it is possible to suppress NFATc1 expression by TPF, its down‐streams c‐Fos and AP‐1 are also down‐regulated. Thus, TPF inhibits c‐Fos–dependent osteoclast‐specific gene expression and bone resorption.^26^ Another importance, c‐Fos pathways crosstalk to activation of inflammatory pathways in osteoclasts, and therefore, TPF may suggest as an important therapy for the treatment of inflammatory bone diseases, like osteoporosis. As suppression of osteoclast function by TPF modulates the disturbance of actin ring production, the expression osteoclast differentiating markers such as cathepsin K, MMP‐9, MMP‐13, OSCAR and TNF‐α are also inhibited by TPF. However, TPF‐mediated osteoclast inhibition needs more further clarification for molecular insight. The present study demonstrated that TPF inhibited osteoclastogenesis by down‐regulation c‐Fos activation, subsequently suppressed the expression of gene related to osteoclastogenesis and attenuated the activation and nuclear translocation of AP‐1 and NFATc1. Our findings suggest that TPF be a potential therapeutic candidate for the treatment of bone diseases associated with excessive bone resorption.

## CONFLICT OF INTEREST

All of the authors clearly declare that they have no competing and commercial interests.

## AUTHOR CONTRIBUTIONS

MAAM designed the study, carried out experimental work on biological investigation, choice of assay methods, critically reviewed the manuscript and proofread. MMHA, MAZS and MAAB assisted in data analysis and interpretation, critically reviewed the manuscript and proofread. This manuscript is not under review elsewhere, and all authors read and approved the final manuscript.

## Data Availability

Data sets generated or analysed during the current study are included in the article.
